# The DNMT1 inhibitor GSK-3484862 mediates global demethylation in murine embryonic stem cells

**DOI:** 10.1186/s13072-021-00429-0

**Published:** 2021-12-15

**Authors:** Nathalia Azevedo Portilho, Deepak Saini, Ishtiaque Hossain, Jacinthe Sirois, Christopher Moraes, William A. Pastor

**Affiliations:** 1 Department of Biochemistry, McGill University, Montreal, QC H3G 1Y6 Canada; 2 Department of Chemical Engineering, McGill University, Montreal, QC H3A 0C5 Canada; 3 The Rosalind and Morris Goodman Cancer Institute, McGill University, Montreal, QC H3A 1A3 Canada

**Keywords:** DNA methylation, DNMT1, GSK-3484862, GSK-3685032, 5-Azacytidine, Decitabine, Demethylation, Whole-genome bisulfite sequencing

## Abstract

**Background:**

DNA methylation plays an important role in regulating gene expression in mammals. The covalent DNMT1 inhibitors 5-azacytidine and decitabine are widely used in research to reduce DNA methylation levels, but they impart severe cytotoxicity which limits their demethylation capability and confounds interpretation of experiments. Recently, a non-covalent inhibitor of DNMT1 called GSK-3484862 was developed by GlaxoSmithKline. We sought to determine whether GSK-3484862 can induce demethylation more effectively than 5-azanucleosides. Murine embryonic stem cells (mESCs) are an ideal cell type in which to conduct such experiments, as they have a high degree of DNA methylation but tolerate dramatic methylation loss.

**Results:**

We determined the cytotoxicity and optimal concentration of GSK-3484862 by treating wild-type (WT) or *Dnmt1/3a/3b* triple knockout (TKO) mESC with different concentrations of the compound, which was obtained from two commercial sources. Concentrations of 10 µM or below were readily tolerated for 14 days of culture. Known DNA methylation targets such as germline genes and GLN-family transposons were upregulated within 2 days of the start of GSK-3484862 treatment. By contrast, 5-azacytidine and decitabine induced weaker upregulation of methylated genes and extensive cell death. Whole-genome bisulfite sequencing showed that treatment with GSK-3484862 induced dramatic DNA methylation loss, with global CpG methylation levels falling from near 70% in WT mESC to less than 18% after 6 days of treatment with GSK-3484862. The treated cells showed a methylation level and pattern similar to that observed in *Dnmt1*-deficient mESCs.

**Conclusions:**

GSK-3484862 mediates striking demethylation in mESCs with minimal non-specific toxicity.

**Supplementary Information:**

The online version contains supplementary material available at 10.1186/s13072-021-00429-0.

## Introduction

DNA methylation is a key regulator of gene expression in mammals [1]. DNA methyltransferases (DNMTs) transfer a methyl group to the fifth carbon of the DNA base cytosine to form 5-methylcytosine. Typically, a high density of DNA methylation at a gene or transposon’s transcriptional start site promotes transcriptional repression [2]. DNMT3A and DNMT3B methylate previously unmodified CpG sites, establishing the pattern of DNA methylation during early embryonic development [3]. DNMT1 in turn methylates the newly synthesized DNA strand after DNA replication. Because DNMT1 preferentially methylates CpGs complementary to, or in the vicinity of, existing CpG methylation, it can maintain CpG methylation patterns through many cycles of cell division [4, 5].

DNA methylation is critical for maintaining silencing of certain genes, particularly genes associated with germline development, and transposons [1, 6, 7]. Aberrant hypermethylation of the promoters of tumor suppressors and other genes occurs in a wide variety of cancers [8]. Consequently, there is high interest in demethylating drugs for both research and therapeutic purposes [9]. Two such drugs are widely used in research and as therapeutics: 5-azacytidine and decitabine (5-aza-2’-deoxycytidine). They were synthesized in the 1960s as potential chemotherapeutics that might interfere with nucleic acid metabolism in rapidly dividing cells [10, 11]. It was subsequently demonstrated that unlike other chemotherapeutics, 5-azacytidine and related compounds induce differentiation of sarcoma cells to myotubes, an activity attributable to apparent demethylating properties [12].

5-Azacytidine and decitabine are cytidine analogs which contain nitrogen at the fifth position of the pyrimidine ring [10, 11]. In cells, decitabine is converted to 5-aza-dCTP and incorporated into DNA during replication [13]. The 5-azacytosine base forms a stable covalent bond with DNMT1, irreversibly inhibiting the enzyme [14]. The resulting DNA–protein adduct is then repaired and DNMT1 is destroyed in the proteasome [15]. Decitabine thus has two modes of activity: it causes cell death via the creation of excessive DNA–protein adducts, and it reduces DNA methylation by the inhibition and destruction of DNMT1 [16]. 5-Azacytidine is converted into a mixture of 5-aza-dCTP and 5-aza-CTP in the cell [17]. As such, it is incorporated into both DNA and RNA, and may cause additional cytotoxicity by interfering with RNA metabolism, methylation, and translation [18, 19].

The cytotoxicity of 5-azanucleosides limits their use as demethylating agents in research. Excessive concentrations of 5-azanucleosides will cause cell death, and thus experiments must be conducted in a narrow window of optimal concentration in which DNMT1 is inhibited, but the cells do not die [9]. At tolerated doses, DNMT1 inhibition may be incomplete, and 5-azanucleoside-treated cells show only modest global reductions in DNA methylation when measured by highly quantitative whole-genome bisulfite sequencing [20, 21]. Even at lower concentrations, the genotoxic effects of 5-azanucleoside make it unclear whether gene expression changes and cellular phenotypes are caused by demethylation or non-specific toxicity. To identify genes regulated by DNA methylation, Hackett and colleagues treated NIH3T3 cells with decitabine for three days and identified 344 upregulated genes [22]. Few of these upregulated genes had heavily methylated promoters and many upregulated genes were involved in immune and stress response, suggesting a response to toxicity rather than demethylation. After 14 days of recovery, 49 genes showed persistent upregulation. These genes were disproportionately enriched for heavily methylated promoters and likely to be direct targets of methylation-mediated silencing. This strategy of treatment and recovery was successful at identifying genes regulated by DNA methylation in NIH3T3s, but carries obvious drawbacks. Any gene remethylated during the recovery period would be missed. Furthermore, researchers studying the role of DNA methylation during a dynamic process cannot necessarily incorporate a treatment and recovery period. A DNA methyltransferase inhibitor lacking non-specific toxicity would be preferable from a research perspective.

Despite their limitations, 5-azanucleosides have remained the best and most widely used drugs for inducing DNA demethylation for the last 40 years. In 2019, GlaxoSmithKline announced the discovery of a non-covalent DNMT1 inhibitor, GSK-3484862 [23]. As of this writing, this compound has been described in three articles. Gilmartin and colleagues determined that GSK-3842364, a racemic mixture including GSK-3484862 and its enantiomer, selectively inhibited DNMT1 with an IC_50_ of 0.4 µM [24]. Five days of treatment of GSK-3842364 induced a dramatic global demethylation of erythroid progenitor cells as measured by mass spectrometry. In addition, GSK-3842364 showed lower cytotoxicity than decitabine and induced transcription of the methylated fetal hemoglobin genes, *HBG1* and *HBG2*, both in erythroid cells in vitro and in a murine model of sickle cell anemia. In the second report, Haggarty and colleagues used GSK-3484862 to inhibit DNMT1 in murine pre-implantation *Dnmt3a/3b* KO embryos [25]. Here, a concentration above 0.35 µM prevents blastocyst formation. At this low concentration, only a 34% global drop in DNA methylation is observed relative to untreated *Dnmt3a/3b* KO embryos; however, the treatment is sufficient to induce a marked increase in *IAP-Ez* transposon expression, consistent with demethylating activity of the compound. Most recently, Pappalardi and colleagues described the discovery of GSK-3484862 in a screen for DNMT1 inhibitors and the characterization of two related compounds, GSK-3685032 and GSK-3830052 [26]. They demonstrated that these inhibitors make simultaneous contact with DNA and the DNMT1 active-site loop and block activity of the enzyme. In leukemia cell lines, these inhibitors cause reduced DNA methylation and increased expression of methylated genes and human endogenous retroviruses (hERVs). Upregulation of interferon response and growth arrest were also observed, which are known to be consequences of hERV upregulation [27]. Finally, Pappalardi and colleagues demonstrated effectiveness of GSK-3685032 in mouse leukemia xenotransplantation models.

Considering the importance of a better DNA methylation inhibitor for the scientific community, we tested the demethylating capacity of GSK-3484862 in murine embryonic stem cells (mESCs), which are ideal for testing methyltransferase inhibitors. When cultured in classic serum + LIF conditions, mESCs have a high global level of DNA methylation [28]. However, while most mammalian cells require some DNA methylation for survival [29], *Dnmt1/3a/3b* triple knockout (TKO) ESCs self-renew and proliferate normally [30]. The set of genes regulated by DNA methylation in mESCs is well characterized, as are individual and compound DNA methyltransferase knockouts [31, 32]. In addition to wild-type (WT) cells, we used *Dnmt1/3a/3b* TKO mESCs because lacking DNA methylation, these cells are suitable to assess non-specific cytotoxicity of GSK-3484862. Our results indicate that GSK-3484862 mediates striking demethylation in mESCs, comparable to what is observed in a complete DNMT1 knockout, with minimal non-specific toxicity.

## Results

### mESCs tolerate high concentrations of GSK-3484862

**Fig. 1:**
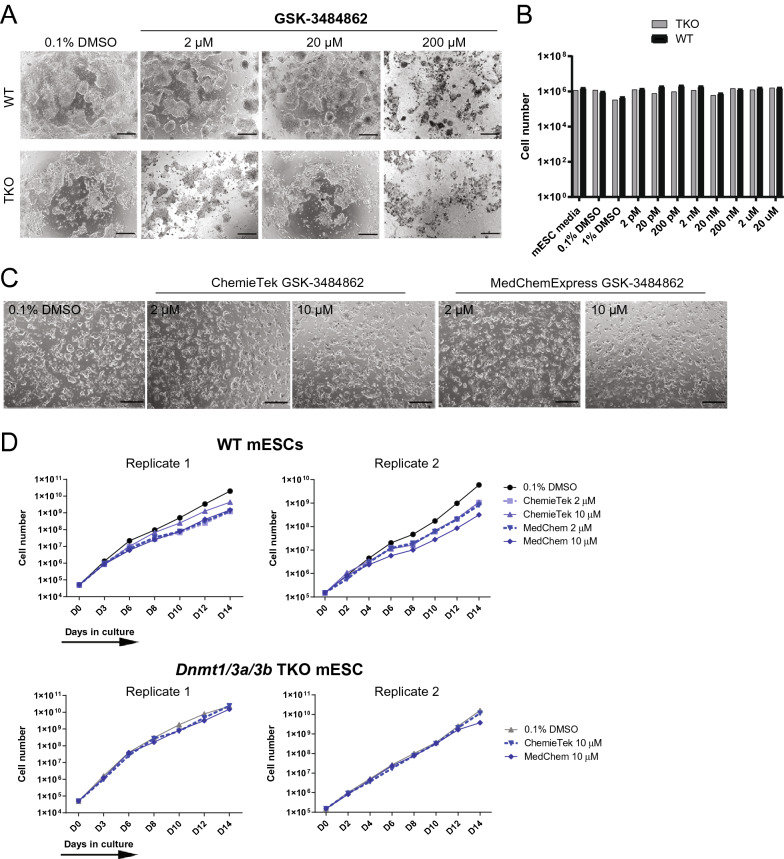
GSK-3484862 inhibitor treatment in mESC. **A** Brightfield images showing morphology of WT and TKO cells after six days of treatment with 0.1% DMSO or GSK-3484862 (2 µM, 20 µM and 200 µM) obtained from ChemieTek. Scale bar = 500 μm **B** Cell numbers after 30,000 WT or TKO mESC were treated with indicated culture conditions for six days. **C** Brightfield images of WT cells after 14 days of treatment with 0.1% DMSO, 2 µM or 10 µM of GSK-3484862 from ChemieTek or MedChemExpress Scale bar= 500 μm. **D** Graphs from two independent experiments show the cell growth of WT or TKO cells passaged and counted every 2–3 days

As of this writing, GSK-3484862 and related inhibitors are not available for sale from GlaxoSmithKline, so we conducted experiments using GSK-3484862 purchased from ChemieTek and MedChemExpress. First, we sought to determine the cytotoxicity and optimal concentration of GSK-3484862 (Fig. 1). WT J1 line and *Dnmt1/3a/3b* TKO mESCs on a J1 background were seeded at low density on gelatin-coated plates. WT mESCs showed classic mESC morphology, dome-shaped colonies with rounded edges, while TKO ESCs showed jagged colony edges when cultured on gelatin. Cells were treated 24 h after plating with concentrations of GSK-3484862 ranging from 2 pM to 200 µM. Substantial precipitation of the drug in media was observed at concentrations equal to or greater than 20 µM. After six days, the morphology of WT and TKO cells treated with GSK-3484862 was similar to that of corresponding control cells that received only DMSO, with mortality only noticeable at 200 µM (Fig. 1A). No differences were observed in cell numbers after 6 days of treatment with GSK-3484862 at concentrations equal to or below 20 µM in WT and TKO cells (Fig. 1B). Consistent with a prior observation [24], treatment with GSK-3484862 resulted in a modest reduction in DNMT1 protein level despite being a non-covalent inhibitor with no measurable effect on cell growth during this time interval (Additional file 1: Fig. S1).

We then evaluated the long-term cytotoxicity and demethylation efficacy of GSK-3484862. WT and TKO cells were treated with 2 µM or 10 µM of GSK-3484862 for 14 days, 10 µM being the highest concentration at which drug precipitation was not observed. WT drug-treated mESCs were indistinguishable from corresponding vehicle-treated controls throughout the 14 days (Fig. 1C). A slight but reproducible decrease in cell growth was observed in GSK-3484862-treated WT cells from day six onwards, whereas the growth rate of TKO cells was constant throughout treatment (Fig. 1D). No difference was observed between compound produced by the two companies (Fig. 1C, D). Altogether, the results indicate that mESCs tolerate GSK-3484862 for up to two weeks at concentrations up to 10 µM.

### Genes repressed by DNA methylation show derepression in GSK-3484862

**Fig. 2:**
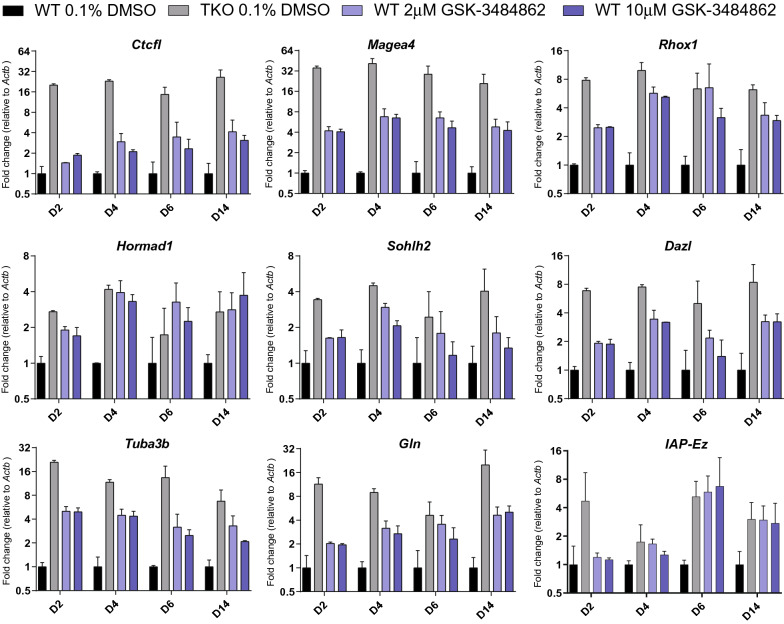
Expression of methylation-regulated transcripts in GSK-3484862 treated mESC. RT-qPCR, normalized to *Actb*, of known methylation-regulated germline genes/transposons in cells treated with GSK-3484862 over the number of days indicated. N = 2–6 biological replicates per sample. Mean and standard error are indicated

We next assayed expression levels of seven genes (*Ctcfl, Magea4, Rhox1, Hormad1, Sohlh2, Dazl, Tuba3b*) and two transposon classes (*Gln, IAP-Ez*) reported to be repressed by DNA methylation in mESCs [31–33]. As expected, all tested transcripts were highly upregulated in TKO cells (Fig. 2, Additional file 2: Table S1). These transcripts were also upregulated after treatment with GSK-3484862, although often to a lesser extent than in TKO cells, potentially reflecting continued expression of DNMT3A and DNMT3B in the WT cells. GSK-3484862 produced by both companies showed similar levels of upregulation (Additional file 2: Table S1). Upregulation of target transcripts was observed in WT cells treated with either 2 µM or 10 µM GSK-3484862, starting as early as after two days of exposure (Fig. 2). All transcripts showed statistically significant upregulation at both concentrations in a one-sided t-test, except for IAP-Ez which showed more gradual upregulation and fell short of statistical significance (Additional file 3: Table S2).

### Severe toxicity from 5-azacytidine or decitabine treatment

**Fig. 3:**
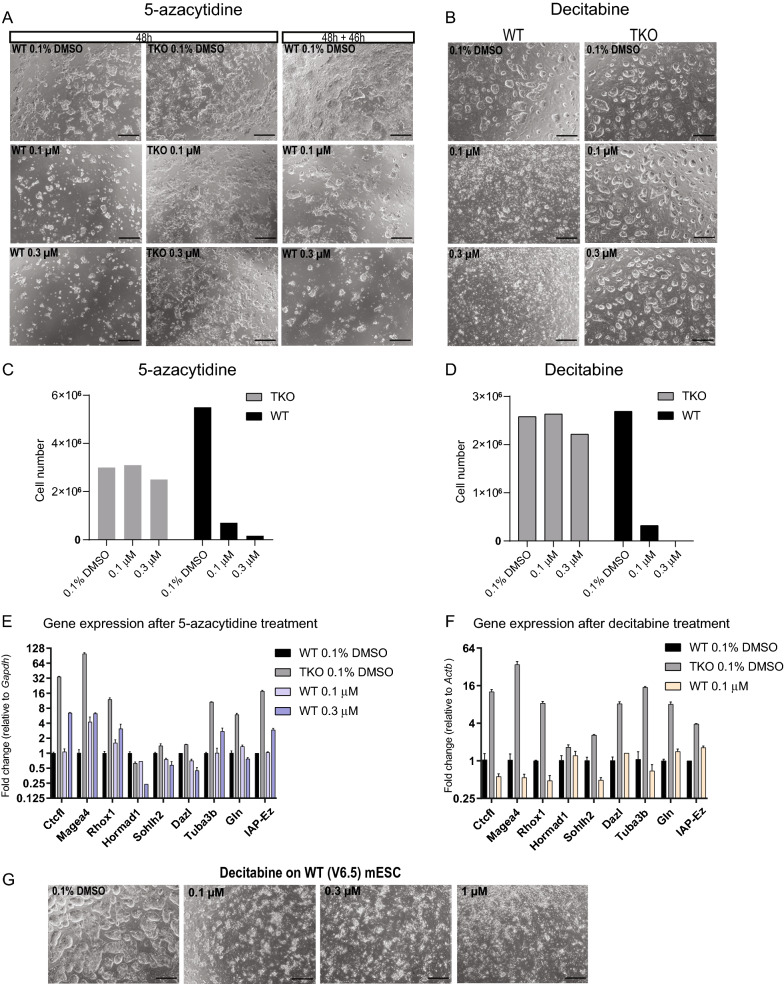
Treatment of mESCs with 5-azanucleosides.** A** Brightfield images of WT and *Dnmt1/3a/3b* TKO mESCs after 48 h of exposure to 0.1% DMSO, 0.1 µM or 0.3 µM 5-azacytidine (left columns) and WT cells 46 h after the end of treatment with 5-azacytidine (right column). Scale bar = 500 μm **B** Brightfield images taken of WT and TKO mESCs after 48 h of exposure to 0.1% DMSO, 0.1 or 0.3 µM decitabine. To enhance survival, these cells were cultured on murine embryonic feeders (MEFs) rather than gelatin, hence the distinct morphology. Scale bar = 500 μm **C, D** Cell numbers after treatment and recovery for 5-azacytidine (**C**) and decitabine (**D**). **E, F** Expression of methylated genes upon 5-azacytidine (**E**) or decitabine (**F**) treatment and recovery, normalized to *Gapdh* and *Actb*, respectively. Mean and standard error of two technical replicates are indicated. **G** V6.5 mESCs after exposure to decitabine. These cells showed similar lethality to J1 WT mESCs

WT and TKO cells were exposed to 5-azacytidine or decitabine for 48 h. Concentrations as low as 0.1 µM of these compounds inflicted widespread cell death in WT cells (Fig. 3A, B). TKO cells, which lack DNMT1 and thus will not form DNMT1-DNA adducts, appeared completely unaffected. After 1–2 days of recovery time, some WT ESC colonies of typical morphology were observed from the 0.1 µM and 0.3 µM 5-azacytidine and the 0.1 µM decitabine treated samples (Fig. 3A–D), and these were used for subsequent analysis.

WT cells treated with 0.3 µM 5-azacytidine showed higher expression of *Tuba3b*, *Ctcfl*, *Magea4 Rhox1* and *IAP-Ez*, while there was no change in the expression of the other transcripts tested (Fig. 3E). Minimal or no upregulation of methylation-repressed transcripts was observed in WT cells treated with decitabine (Fig. 3F.)

Comparable decitabine-mediated toxicity was observed for WT mESCs from the genetic hybrid V6.5 line, confirming that this was not a cell line specific effect (Fig. 3G).

### Whole-genome bisulfite sequencing showed dramatic demethylation in mESCs after GSK-3484862 treatment

**Fig. 4:**
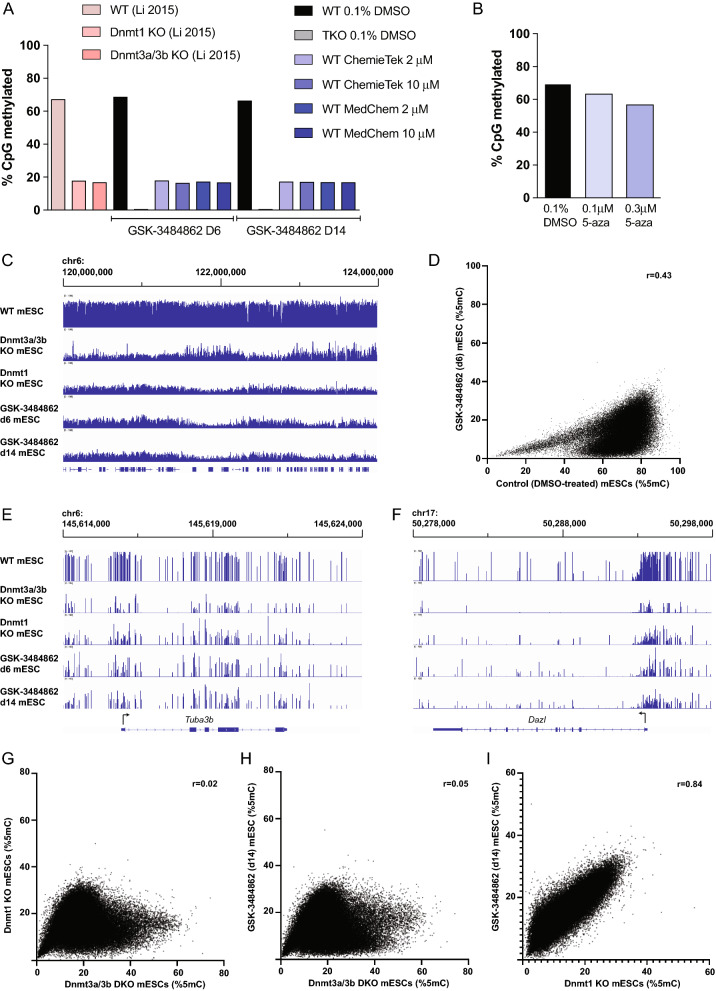
CpG methylation reduction after treatment with GSK-3484862 or 5-azacytidine. **A** Global CpG methylation levels of WT cells treated with 0.1% DMSO, *Dnmt1/3a/3b* TKO cells treated with 0.1% DMSO, and WT cells after six or 14 days of treatment with 2 µM or 10 µM GSK-3484862. Methylation levels of published *Dnmt1*^*−/−*^* and Dnmt3a*^*−/−*^*3b*^*−/−*^ knockout cells are shown in comparison [31]. **B** Methylation levels in WT mESCs after treatment with 0.1% DMSO, 0.1 µM or 0.3 µM 5-azacytidine. **C** DNA methylation over a 4-MB region over chromosome 6 in samples indicated. The first three samples are from published data [31]. Height of bar indicates extent of CpG methylation in a region, from 0 to 100%. **D** DNA methylation level of 10 kb regions of the genome are indicated, with each region plotted as a single point. **E**, **F** DNA methylation level over the *Tuba3b* and *Dazl* genes. **G**, **I** DNA methylation level of 10 kb regions of the genome are plotted for the samples shown, with Pearson correlation coefficient indicated. The *Dnmt1*^*−/−*^ and *Dnmt3a*^*−/−*^*3b*^*−/−*^ data are from published data [31]

Whole-genome bisulfite sequencing, even at low sequencing depth, provides an accurate quantitative estimate of total CpG methylation [34]. Based on low-depth sequencing, WT mESCs showed near 70% CpG methylation, dropping below 18% after six or 14 days of treatment with GSK-3484862, for both companies and at both concentrations (Fig. 4A). Mapping statistics and conversion efficiencies are shown in Additional file 4: Table S3. Cells treated with 5-azacytidine showed far more modest reductions in 5-methylcytosine (Fig. 4B), but they may have regained some DNA methylation during the 46 h recovery period after treatment.

We then sequenced three samples (d14 control mESCs, d6 and d14 2 µM GSK-3484862 mESCs) at higher depth and compared with published DNA methylation data generated by Li and colleagues [31]. By splitting the genome into 10 kb bins and plotting the methylation level of each bin, we confirmed high similarity of our control mESCs to published WT mESCs (Additional file 1: Fig. S2A), and similarity of the d6 and d14 GSK-3484862 treated samples to each other (Additional file 1: Fig. S2B). We observed a dramatic and largely even loss of DNA methylation upon inhibitor addition (Fig. 4C, D). Appropriately, demethylation was observed at methylation-regulated genes upregulated in Fig. 2 (Fig. 4E, F).

We then compared the GSK-3484862-treated mESCs with the *Dnmt1*^*−/−*^ and *Dnmt3a*^*−/−*^* 3b*^*−/−*^ mESCs. The overall methylation level of GSK-3484862-treated cells was virtually identical to that of either methylation mutant (Fig. 4A). Despite the similar global levels of methylation, there were noticeable differences in the local methylation patterns of the *Dnmt1*^*−/−*^ and *Dnmt3a*^*−/−*^* Dnmt3b*^*−/−*^ mESCs, with GSK-3484862-treated cells showing close similarity to *Dnmt1*^*−/−*^ mESCs (Fig. 4C). Indeed, comparing methylation level over 10 kb bins, we found striking similarity of the GSK-3484862-treated cells with *Dnmt1*^*−/−*^ mESCs and weak similarity of either of these cell types with *Dnmt3a*^*−/−*^* Dnmt3b*^*−/−*^ cells (Fig. 4G–I). IAP-Ez elements, which retain a high degree of DNA methylation in the *Dnmt3a*^*−/−*^* Dnmt3b*^*−/−*^ mutants because of their high propensity to attract DNMT1 [25], likewise show dramatic methylation loss in *Dnmt1*^*−/−*^ or GSK-3484862-treated mESCs (Additional file 1: Fig. S2C). In total, methylation mapping supports the conclusion that GSK-3484862 treatment produces a drop in DNA methylation consistent with selective and near complete inhibition of DNMT1.

## Discussion

From a research perspective, GSK-3484862 shows a great deal of promise. 5-Azanucleosides have substantial non-specific toxicity and work within a narrow concentration band. We observed reactivation of some methylated genes after treatment with 0.3 µM 5-azacytidine, but very few cells survived at this concentration, while 0.1 µM 5-azacytidine was inadequate to reactivate methylated genes. Furthermore, we had to allow two days for colonies of surviving cells to emerge in culture. By contrast, 2 µM or 10 µM GSK-3484862 reactivated methylated gene expression and produced only modest reduction in growth, most noticeable between days six and 10 of treatment. This reduced growth may reflect specific activity of the compound. When mESCs undergo demethylation mediated by treatment with MEK inhibitor, Glycogen Synthase Kinase 3 inhibitor and high concentration of ascorbic acid, they have a burst of transposon expression and undergo reconfiguration of heterochromatin state approximately during this interval [33].

DNA methylation of the GSK-3484862-treated mESCs never fell below 16% regardless of the dosage used or time of treatment, and methylated genes were not reactivated to the extent observed in the TKO cells. This likely reflects the high level of DNMT3A and DNMT3B activity in mESCs, as evidenced by the similar level of DNA methylation in published *Dnmt1* KO mESCs [31]. The *Dnmt1* deficient or inhibited cells potentially reach an equilibrium in which methylation is constantly added by DNMT3A and DNMT3B and lost through replication and Tet-protein activity. Other cell types may respond to *Dnmt1* inhibition differently. Most somatic and cancer cells do not express such high levels of the de novo DNA methyltransferases and may not be able to maintain such high levels of DNA methylation in the absence of *Dnmt1* activity. At the same time, somatic or cancer cells may not survive dramatic DNA methylation loss [29, 35]. *Dnmt1* deficient and *Dnmt1/3a/3b* TKO mESCs are not viable upon differentiation, and DNMT1 becomes essential after uterine implantation [6, 30]. This shift may reflect the fact that mESCs depend heavily on TRIM28 to silence transposons, but upon differentiation of mESCs or uterine implantation of embryos, DNA methylation gains importance for transposon repression [7, 32, 36, 37]. Thus, researchers working with other cell types may well observe specific toxicity at lower doses, and indeed GSK-3484862 and related compounds show a striking effect in leukemias [26]. We also cannot rule out that non-specific toxicity may occur in some cell types, a result suggested by the GSK-3484862’s ability to halt mouse development at relatively low concentrations [25]. Nonetheless, this novel DNA methyltransferase inhibitor appears to be a substantial improvement over 5-azanucleosides and a promising research tool.

## Conclusions

GSK-3484862 mediates dramatic demethylation in murine embryonic stem cells. With regard to both activation of methylated genes and non-specific toxicity, GSK-3484862 performs far better than 5-azanucleosides.

## Methods

### Cell culture

Mouse embryonic stem cells (mESC) lines used in this study included *Dnmt1/3a/3b* triple knockout [30] on a J1 (129S4/SvJae) background, as well as WT J1 and V6.5 (C57BL/6 × 129S4/SvJae) lines. Cells were cultured in Knockout-DMEM (Gibco) with 15% ES qualified FBS (Gibco), ESGRO mouse LIF 1000U/mL (Millipore), 1X PenStrep (Invitrogen), 1X Glutamax (Invitrogen), 55 µM b-mercaptoethanol (Invitrogen), 1X non-essential amino acids (Invitrogen) and 1X Primocin (Invivogen). Cells were maintained at 37 °C and 5% CO_2_ on dishes coated with 0.1% gelatin (Millipore) unless otherwise stated. Experiments in which mESCs were plated on a layer of mitomycin C-treated mouse embryonic fibroblasts (MEFs) (ThermoFisher Scientific) are explicitly stated.

### Determining GSK-3484862 toxicity

An assay to determine the optimal concentration and toxicity of GSK-3484862 (ChemieTek) was performed using J1 WT and DNMT TKO mESCs. 30,000 cells were seeded in 24-well plates pre-coated with 0.1% gelatin. The next day, medium was changed to fresh mESC medium or medium containing DMSO (0.1% or 1%) for the following concentrations of GSK-3484862: 2 pM, 20 pM, 200 pM, 2 nM, 20 nM, 200 nM, 2 µM, 20 µM (in 0.1% DMSO) and 200 µM (in 1% DMSO). The medium was refreshed every day for the next six days, after which cell morphology was assessed, followed by cell dissociation with 0.05% Trypsin–EDTA (Gibco) for cell counting.

Next, the demethylation efficacy and long-term cytotoxicity of GSK-3484862 from two companies, ChemieTek and MedChemExpress, was evaluated in duplicate experiments. To improve solubility, after resuspension in DMSO, GSK-3484862 was subjected to ultrasonication. GSK-3484862 from both companies were sonicated for 6 min at 42 kHz in an ultrasonic water bath (Sper Scientific). Still, drug precipitation was observed for concentrations at or above 20 µM in media, and therefore an upper concentration of 10 µM was chosen. WT and DNMT TKO cells were seeded in 12-well plates pre-coated with 0.1% gelatin and had 0.1% DMSO, 2 µM or 10 µM GSK3484862 added in medium from day zero. The medium was refreshed every day and cells were counted using the Countess II FL instrument and passaged every 2–3 days for the next 14 days.

### 5-Azacytidine assay

We found that WT mESCs died if treated with 5-azacytidine immediately after plating (data not shown). Therefore, we plated cells at low density (1.4 × 10^4^ cells/ cm^2^) and added 0.1% DMSO, 0.1 µM or 0.3 µM of the drug 24 h later. WT and DNMT TKO cells were exposed to 5-azacytidine for 48 h, with a media change at the 24 h timepoint to replenish 5-azacytidine. The cultures were then maintained in media without the drug for 46 h. Cells were then dissociated with 0.05% Trypsin–EDTA, counted, and collected for subsequent analysis. Because of their higher survival and consequent higher density, DNMT TKO cells were harvested after only 24 h recovery time.

### Decitabine assay

Concentrations of decitabine as low as 0.1 µM proved lethal to WT mESCs plated on gelatin (data not shown), so mESCs were seeded at 1.4 × 10^4^ cells/ cm^2^ on a monolayer of MEF feeder cells to enhance survival. 24 h later, 0.1% DMSO or decitabine at concentrations of 0.1 µM, 0.3 µM or 1 µM was added and cells were treated for 48 h, with a media change at the 24 h timepoint. Cells were allowed to recover for another 24 h and then harvested. Because of the low viability of the WT J1 line mESCs, the experiment was repeated with V6.5 mESCs, a robust line on a hybrid genetic background (C57BL/6 × 129S4/SvJae).

### Cell imaging and counting

All microscopy images were acquired with an EVOS M5000 (Invitrogen) and cell numbers were counted using Countess II FL Automated Cell Counter (Invitrogen). Images were processed using Adobe Photoshop and ImageJ, and graphs were created using GraphPad Prism software.

### DNA and RNA extraction

Cell pellets were snap frozen and kept at –80 °C until extraction. RNA and DNA from GSK-3484862 and 5-azacytidine treated samples were isolated simultaneously using the AllPrep DNA/RNA kit (Qiagen), whereas only RNA was isolated from decitabine treated samples using the RNAzol-RT total RNA protocol (Sigma). DNA and RNA concentrations were measured with the Qubit dsDNA and RNA HS Assay Kits, respectively (ThermoFisher).

### Western blot analysis of DNMT1 protein levels in cells treated with GSK inhibitor

To validate the specificity of the DNMT1 antibody and DNMT1 deletion in the DNMT TKO, both J1 WT and DNMT TKO cells were cultured in a 6-well plate pre-coated with 0.1% gelatin. Medium was refreshed every day until cells were 70–80% confluent. Cells were then dissociated with 0.05% Trypsin–EDTA and pellets were snap frozen for protein extraction.

To determine the effect of GSK-3484862 on DNMT1 protein levels, J1 WT cells were seeded in 12-well plates pre-coated with 0.1% gelatin and treated with medium supplemented with 0.1% DMSO, 2 µM or 10 µM of GSK-3484862 (ChemieTek). The medium was refreshed every day for the next four days, with cells being passaged two days post-plating. After four days of treatment, cells were dissociated with 0.05% Trypsin–EDTA and pellets were snap frozen and stored at -80 °C for subsequent protein extraction.

Protein was extracted in ice cold RIPA lysis buffer, supplemented with protease inhibitors (1 mM phenylmethylsulfonyl fluoride, 10mM sodium fluoride and 1 mM sodium orthovanadate). Cell samples were then exposed to 5 cycles of freeze–thaw using liquid nitrogen to ensure complete lysis. Protein lysate concentrations were measured using a Bradford Assay and 30 μg of protein were subjected to a 6%-12% gradient SDS-PAGE. The resolved proteins were transferred to a polyvinylidene fluoride (PVDF) membrane and blocked using 5 mL of LI-COR Odyssey Blocking Buffer for 1 h at room temperature. Primary antibodies (DNMT1 (Santa-Cruz H-300) 1:1000 dilution and Histone H3 (Abcam ab10799) 1:10,000 dilution) were diluted in Odyssey Blocking Buffer supplemented with 0.15% Tween-20 and incubated overnight at 4 °C. Membranes were then washed three times for 5 min each in PBS supplemented with 0.1% Tween-20 and incubated in secondary antibodies (LI-COR IRDye 680RD and LI-COR IRDye 800CW, 1:20,000 dilution) for 1 h at room temperature. Membranes were then washed three times for 5 min each in PBS 0.1% Tween and left in PBS before imaging on the LI-COR imaging system. Fiji was used to quantify fluorescent band intensities of DNMT1, which were normalized to band intensities of Histone H3.

### Quantitative RT-PCR

500 ng of RNA was used for cDNA synthesis using Froggabio SensiFAST cDNA synthesis kit and following the manufacturer's instructions. The qPCR reaction was performed using PowerUp SYBR green mix (Invitrogen) containing cDNA generated from the equivalent of 5 ng RNA and 0.5 μM of primer mix (forward and reverse) in a final reaction of 6 µL per duplicate. Quantification and analysis were performed with the QuantStudio5 instrument (Applied Biosystems), and the cycling conditions were: (50 °C 2 min, 95 °C 20 s, 55x (95 °C 3 s, 60 °C 30 s), 95 °C 1 s). The following primers were used:

*Actin*: F:5′-ACTGGGACGACATGGAGAAG-3 ′ R:5′-GGGGTGTTGAAGGTCTCAAA-3′, *Gapdh*: F:5 ′-CATCAAGAAGGTGGTGAAGC-3′ R:5′-GGGAGTTGCTGTTGTAAGTCG 3′, *Tuba3b*: F:5′-AGGAAGATGCAGCCAACAATTA-3′ R:5′-TGCACAGATCGGCCAGTTT-3′, *Ctcfl*: F:5′-GCCTTCAGCATTGCGTGAC-3′ R:5′-AGCAGGTGAAAATGTATCCGC-3′, *Magea4*: F:5′-GTCTCTGGCATTGGCATGATAG-3′ R:5′-GCTTACTCTGAACATCAGTCAGC-3′, *Rhox1*: F:5′-CCGGTTTTCTGGAGTATGAGAGA-3′ R:5′-CCAGCCGTTTTCTGTCTTGTG-3′, *Hormad*1: F:5′-TGAAAACTCTGGAGCTTCTGAAA-3′ R:5′-ACTGACTAACTGTTCAACCTGACA-3′, *Sohlh*2: F:5′-CCATCGAGCTGTTCCTTCCA-3′ R:5′-GGAATACACGTTCAGGCCCC-3′, *Dazl*: F:5′-GTGGCTTCTGCTCCACCTTCG-3′ R:5′- CCTTGACTTGTGGTTGCTGA-3′, *Gln*: F:5′-CGTAAGGACCCTAGTGGCTG-3′ R:5′- GCACTCACTCTTCTTCACTCTG-3′, *IAP-Ez*: F:5′-AAGCAGCAATCACCCACTTTGG-3’ R:5’- CAATCATTAGATGYGGCTGCCAAG-3’. The *Dazl, Gln* and *IAP-Ez* primers were taken from published sources [32].

### Analysis of qRT-PCR data

To calculate which genes showed statistically significant upregulation relative to control cells, expression of each gene in the DNMT TKO or GSK-3484862 cells was normalized to the control or control lines in the same experimental replicate and time-course. Data from cells treated with GSK-3484862 from the two manufacturers (ChemieTek and MedChemExpress) were combined. A paired, one-tailed t-test was conducted, pairing control cells with treated cells for each time point and calculating one p-value for each GSK-3484862 concentration across all timepoints.

### Whole‑genome bisulfite library preparation

Genomic DNA (500 ng) with spiked-in lambda DNA (1.25 ng) (NEB) was sheared using an M220 ultrasound sonicator (Covaris) to an average size of 350 bp. The size of the DNA fragments was confirmed by electrophoresis on a 1.5% agarose gel. 200 ng of sheared DNA was subjected to bisulfite conversion using EZ DNA Methylation-Gold Kit (Zymo Research) as per manufacturer’s instructions. Libraries were prepared using 50–100 ng of bisulfite-converted DNA and the Accel-NGS Methyl-Seq DNA Library prep kit (Swift Biosciences) according to manufacturer's instructions, with 8–11 cycles of PCR-amplification. The integrity of the libraries was assessed using agarose gel electrophoresis.

Libraries were sequenced on an Illumina HiSeq and NovaSeq 6000 instruments at the Michael Smith Genome Sciences Centre at BC Cancer and the Centre for Applied Genomics (SickKids), respectively.

### Whole-genome bisulfite sequencing analysis

Raw reads were quality checked with FastQC and adapters at either end were trimmed with the Trim Galore! (v0.6.6) software. Reads were then aligned to the mm10 reference genome using BSMAPz (v1.1.3). In cases where a given read had multiple equally possible alignments, the read was aligned to at most 500 regions at random. The level of DNA methylation over a given cytosine was calculated using the methratio.py script, with the -r flag. Overall level of DNA methylation for a given condition was calculated as the arithmetic mean of all cytosine bases in CpG context. Methylation calls of all cytosines in CpG context were used to generate bigwig tracks. Briefly, the methylation call files from the methratio.py script were converted to bedGraph file format, with the last column reporting the level of methylation over that cytosine as a percentage; in cases where no methylation was observed for a given cytosine, the value was increased by 1% so that it would appear visible on IGV. Finally, the bedGraphToBigWig command was used to create bigwig tracks which were visualized on IGV.

### Scatterplot and violin plot analysis

The mm10 reference genome was split into 10 kb non-overlapping bins and the average CpG methylation over each bin was calculated using bedops (v2.4.39). Pearson correlation coefficient between two conditions was calculated using the cor.test function in the ggpubr package. Annotation of transposable elements to the mm10 genome was obtained from a gtf file (http://labshare.cshl.edu/shares/mhammelllab/www-data/TEtranscripts/TE_GTF/—accessed 15th November 2021) curated from RepeatMasker. For analysis over IAPEz-int elements, we only considered full length transcripts which we defined as being at least 6 kb in length. CpG methylation over repeat elements is reported as the mean methylation of all CpGs over those regions.

## Supplementary Information

**Additional file 1: Figure S1. A.** Western blot for DNMT1 as well as H3 loading control in WT and *Dnmt1*^-/-^ *3a*^*−/−*^ *3b*^*−/−*^ mESCs. Note loss of DNMT1 in the triple knockout cells, indicating antibody specificity. **B.** Western blot for DNMT1 as well as H3 loading control in WT mESCs and mESCs treated with the indicated concentration of GSK-3484862 for four days. Quantitation of the DNMT1 band, relative to the H3 loading control, is indicated. **Figure S2. A., B.** DNA methylation level of 10-kb regions of the genome are indicated, with each region plotted as a single point. **C.** A violin plot showing the overall methylation level of the 812 full length *IAP-Ez* elements (> 6 kb long) in the cell types indicated. Each *IAP-Ez* element is represented as a single point on the violin plot.

**Additional file 2: Table S1**. qRT-PCR data for all samples.

**Additional file 3: Table S2.** P-values for upregulation of indicated genes and transposons.

**Additional file 4: Table S3.** Mapping and conversion statistics for Whole Genome Bisulfite Sequencing data used in this publication.

## Data Availability

All whole-genome bisulfite sequencing data have been deposited to the Gene Expression Omnibus database under the Accession Number GSE184116.
