# Supplementary material for: The DNMT1 inhibitor GSK-3484862 mediates global demethylation in murine embryonic stem cells
Source: Epigenetics Chromatin. 2021 Dec 15;14:56. doi: 10.1186/s13072-021-00429-0 (PMC8672470; doi:10.1186/s13072-021-00429-0)
Supplement: Supplementary file 1 — Additional file 1: Figure S1. A. Western blot for DNMT1 as well as H3 loading control in WT and Dnmt1-/- 3a−/− 3b−/− mESCs. Note loss of DNMT1 in the triple knockout cells, indicating antibody specificity. B. Western blot for DNMT1 as well as H3 loading control in WT mESCs and mESCs treated with the indicated concentration of GSK-3484862 for four days. Quantitation of the DNMT1 band, relative to the H3 loading control, is indicated. Figure S2. A., B. DNA methylation level of 10-kb regions of the genome are indicated, with each region plotted as a single point. C. A violin plot showing the overall methylation level of the 812 full length IAP-Ez elements (> 6 kb long) in the cell types indicated. Each IAP-Ez element is represented as a single point on the violin plot. [file 13072_2021_429_MOESM1_ESM.pdf]

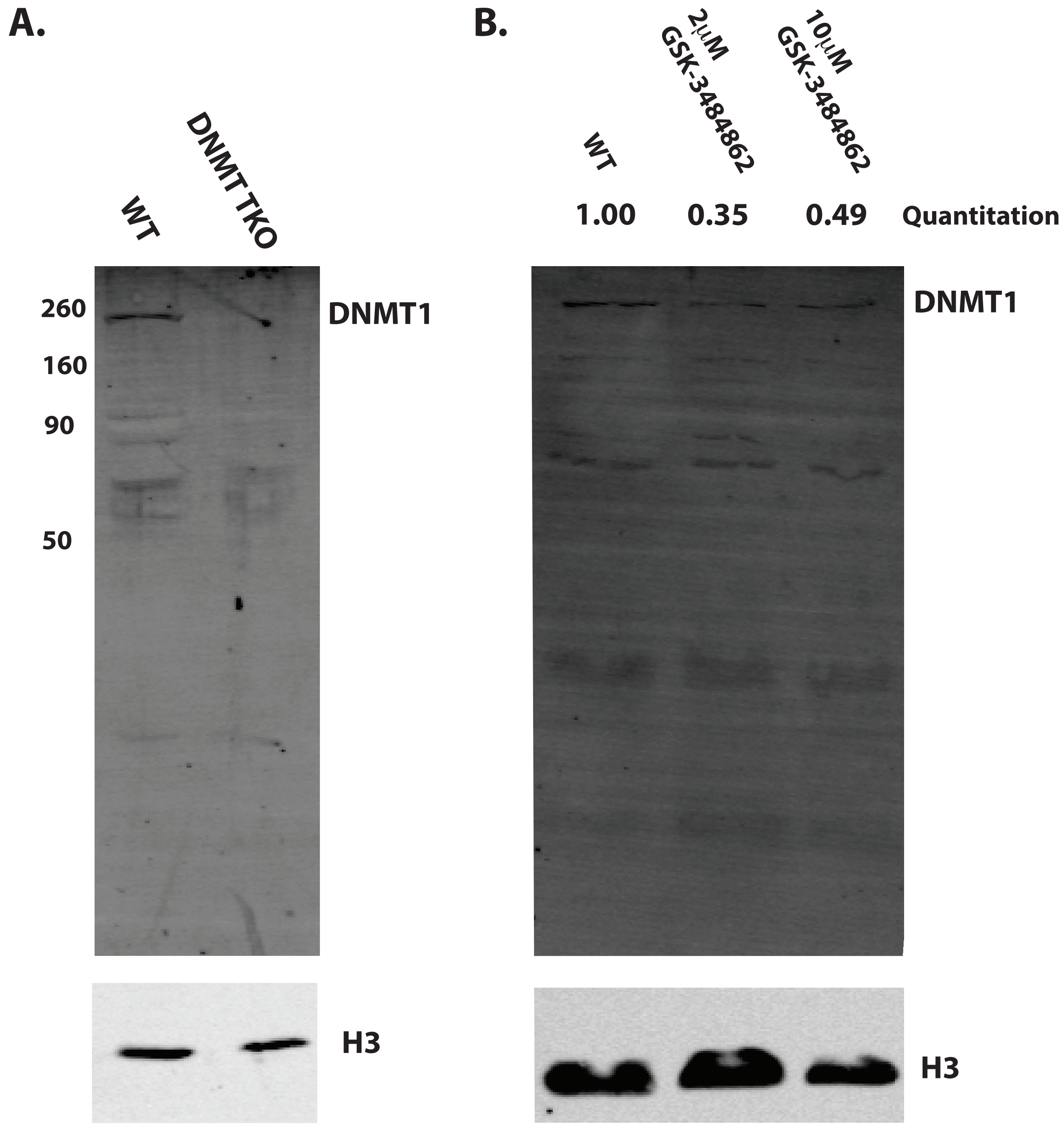

Supplementary Figure S1. A. Western blot for DNMT1 as well as H3 loading control in WT and *Dnmt1*$^{-/-}$ *3a*$^{-/-}$ *3b*$^{-/-}$ mESCs. Note loss of DNMT1 in the triple knockout cells, indicating antibody specificity. **B.** Western blot for DNMT1 as well as H3 loading control in WT mESCs and mESCs cells treated with the indicated concentration of GSK-3484862 for four days. Quantitation of the DNMT1 band, relative to the H3 loading control, is indicated.

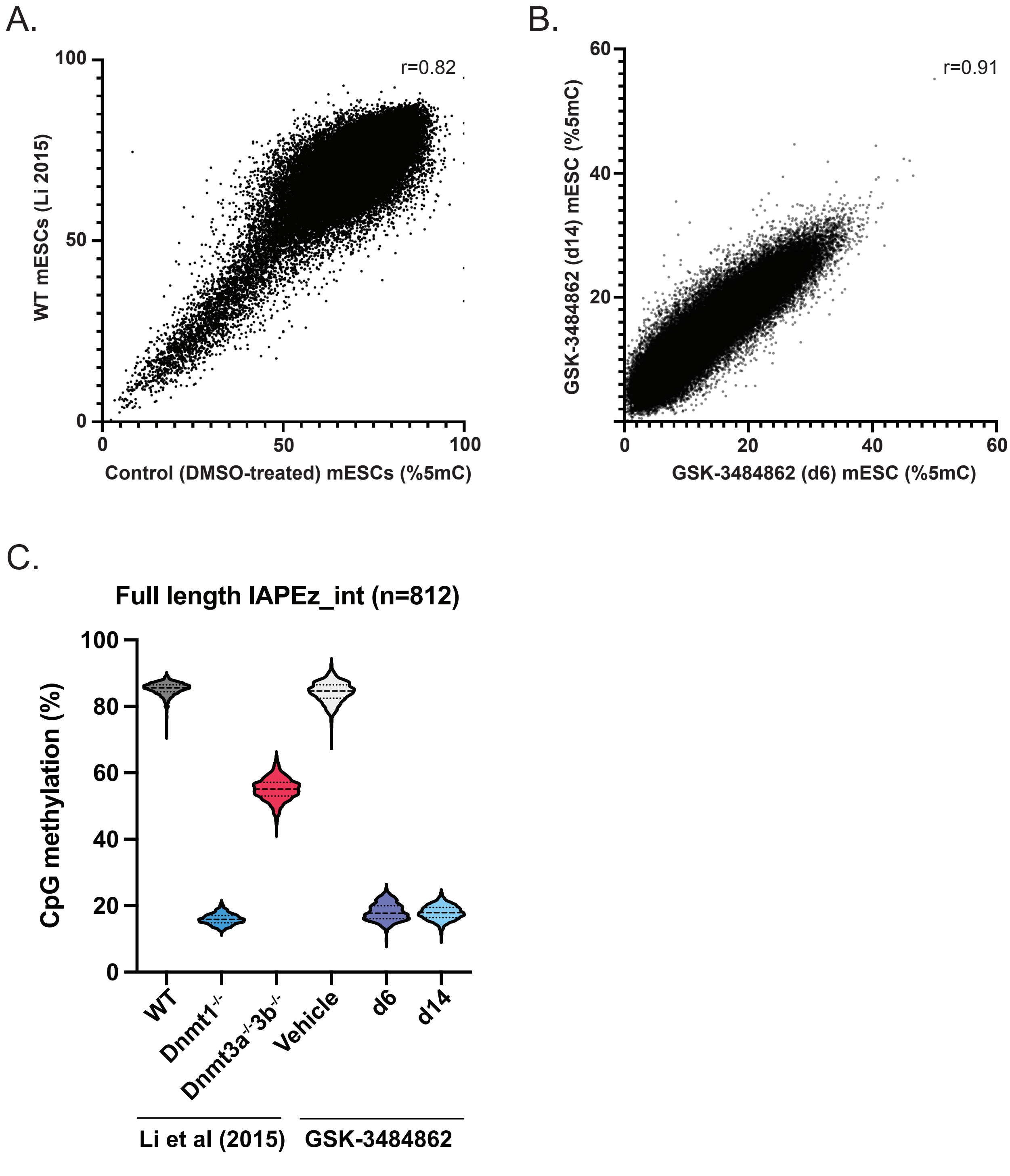

Supplementary Figure S2. A., B. DNA methylation level of 10kb regions of the genome are indicated, with each region plotted as a single point. **C.** A violin plot showing the overall methylation level of the 812 full length *IAP-Ez* elements (>6kb long) in the cell types indicated. Each *IAP-Ez* element is represented as a single point on the violin plot.
